# Clinical characteristics and changing trajectories of esophageal cancer and gastric cancer in China from 2010 to 2019: An analysis of a hospital-based database of 24,327 patients

**DOI:** 10.3389/fonc.2023.1126841

**Published:** 2023-03-13

**Authors:** Qiang Wang, Xiaorui Zhang, Yuxin Zhong, Shijing Wei, Li Li, Wenqiang Wei, Fen Liu, Yong Li, Shaoming Wang

**Affiliations:** ^1^Department of Anesthesiology, National Cancer Center/National Clinical Research Center for Cancer/Cancer Hospital, Chinese Academy of Medical Sciences and Peking Union Medical College, Beijing, China; ^2^Department of Epidemiology and Biostatistics, School of Public Health, Capital Medical University, Beijing, China; ^3^Department of Pancreatic and Gastric Surgery, National Cancer Center/National Clinical Research Center for Cancer/Cancer Hospital, Chinese Academy of Medical Sciences and Peking Union Medical College, Beijing, China; ^4^Medical Records Department, National Cancer Center/National Clinical Research Center for Cancer/Cancer Hospital, Chinese Academy of Medical Sciences and Peking Union Medical College, Beijing, China; ^5^National Central Cancer Registry, National Cancer Center/National Clinical Research Center for Cancer/Cancer Hospital, Chinese Academy of Medical Sciences and Peking Union Medical College, Beijing, China; ^6^Department of Thoracic Surgery, National Cancer Center/National Clinical Research Center for Cancer/Cancer Hospital, Chinese Academy of Medical Sciences and Peking Union Medical College, Beijing, China; ^7^Department of Thoracic Surgery, National Cancer Center/National Clinical Research Center for Cancer/Hebei Cancer Hospital, Chinese Academy of Medical Sciences, Langfang, China

**Keywords:** esophageal cancer, gastric cancer, subsite, histological subtype, comorbidity

## Abstract

**Purpose:**

This analysis aimed to investigate the clinical characteristics and changing trajectories of gastric cancer (GC) and esophageal cancer (EC).

**Methods:**

We collected data from a large cancer hospital in Beijing, China, from 2010 to 2019. Joinpoint regression was used to analyze the trends of histological characteristics and comorbidities.

**Results:**

From 2010 to 2019, there were a total of 10,083 EC patients and 14,244 GC patients. Patients were mainly men and diagnosed at 55-64 years old. Metabolic comorbidity was the most common comorbidity, with hypertension being predominant. The percentages of stage I showed significant increases for EC [average annual percent change (AAPC): 10.5%] and GC (AAPC: 9.7%) patients. We also observed an increasing trend of EC and GC patients over 65 years old. For EC patients, esophageal squamous cell carcinoma (93.1%) remained as the prioritized subtype, and the middle third of the esophagus was the most common site. EC patients with three or more comorbidities increased from 0.1% to 2.2% (AAPC, 27.7%; 95% CI, 14.7% to 42.2%). For GC patients, adenocarcinoma accounts for 86.9% of the total cases, and cardia was the most common site. The ulcerative comorbidity rate decreased from 2.0% to 1.2% (AAPC, −6.1%; 95% CI, −11.6% to −0.3%).

**Conclusion:**

ESCC remained as the prioritized histological subtype, and the middle third of the esophagus was the most common site of EC. The majority of GC patients had adenocarcinoma, and the cardia was the most common site. There was an increasing trend of patients diagnosed at stage I. These findings provide scientific evidence to guide future treatment.

## 1 Introduction

Esophageal cancer (EC) and gastric cancer (GC) are common malignant tumors of the digestive tract. According to estimates from GLOBOCAN in 2020, EC and GC rank as the fifth and seventh most common cancers. Worldwide, an estimated 1.7 million new cancer cases and almost 1.3 million cancer deaths of EC and GC occurred in 2020 (1). Due to their close anatomical location, EC and GC share some common risk factors and epidemiological characteristics. Meanwhile, the screening method for upper gastrointestinal tract cancer (UGIC), including EC and GC, is endoscopic examination in China (2). Both EC and GC can be screened by a single endoscopic examination. In addition, EC and GC also demonstrate histological heterogeneity and have various histologic features (3–5). Esophageal squamous cell carcinoma (ESCC) and esophageal adenocarcinoma (EAC) are the most common histological subtypes of EC. The common subsite of EC was classified as the upper, middle, and lower third of the esophagus. Approximately 90% of GCs are adenocarcinomas, and GC can be subdivided into two main subsites: gastric cardia cancer (GCC) and gastric non-cardia cancer (GNCC). In addition, patients with EC or GC often have one or more comorbidities. The presence of comorbidities increases the disease burden and influences the prevention, detection, treatment decisions, and outcomes (6, 7).

Given the heavy disease burden associated with EC and GC, the Chinese government has launched some screening programs, including two cancer screening and early detection programs in rural areas in 2005 and 2007, respectively, and the Cancer Screening Programs in Urban China launched in 2012 (8). Over the past decade, marked advances have been made in the Chinese cancer prevention and control of EC and GC (9). However, cancer remains a heavy burden in China, and the new EC and GC cases in China account for 53.7% and 43.9% of the world’s new cases, respectively (1).

Although the need to understand the clinical characteristics of EC and GC is well recognized, research conducted on the trajectories of histologic subtypes, subsites, and comorbidity patterns in patients with EC or GC is still limited. To improve the diagnosis and treatment of EC and GC, clinical and pathological characteristics such as histologic subtypes, subsites, and comorbidity patterns need to be considered. This analysis aimed to assess the long-term changing trajectories of EC and GC overall and by sex, age group, histological subtypes, subsites, and comorbidity patterns. The identified changing trajectories in our analysis may help us understand the changing trends and the underlying etiological mechanisms and provide scientific data support for the current cancer diagnosis and treatment in China.

## 2 Materials and methods

### 2.1 Study design and participants

We collected clinical and pathological data from inpatients with EC or GC from the Cancer Hospital, Chinese Academy of Medical Sciences in Beijing, China, from 2010 to 2019, which is the largest cancer hospital in Asia, and enrolled patients across China. Patients with double cancer of EC or GC were included in the analysis for gastric and esophageal cancers. We used the 10th edition of the International Classification of Diseases (ICD-10) for case identification: esophageal cancer (C15.0–C15.5, C15.8–C15.9) and gastric cancer (C16.0–C16.6, C16.8–C16.9). If the site classification was not in the ICD-10 code, it was not included in the analysis. Cases with missing information on the year of diagnosis or age at diagnosis were excluded. Our final analytic sample included 10,083 EC patients and 14,244 GC patients. The study was approved by the Institutional Review Board of the Cancer Hospital, Chinese Academy of Medical Sciences (Ethical approval number: 20/386-2582, **Supplementary File 1**).

### 2.2 Data extraction

We extracted individual patient-level and tumor-related data for all cases. Patient-level data included their age at diagnosis (<55, 55–64, ≥65 years), sex, year of diagnosis, and the type of comorbidities. Tumor-related data included the stage of cancer at diagnosis, cancer subsites, and histologic subtypes. The comorbidity patterns were classified according to the type of comorbidities, including ulcerative comorbidities, inflammatory comorbidities, metabolic comorbidities, and polyp comorbidities. Ulcerative comorbidities include duodenal and gastric ulcer. Inflammatory comorbidities include ulcerative colitis, appendicitis, atrophic gastritis, and gastritis. Metabolic comorbidities include hypercholesterolemia, hypertension, hyperlipidemia, and diabetes. Polyp comorbidities include intestinal polyps, esophageal polyps, and gastric polyps. The cancer stages were reclassified according to the Tumor–Node–Metastasis staging system (7th edition) maintained by the American Joint Committee on Cancer (AJCC). Cancer subsites and histologic subtypes were identified by ICD-10. GCC was defined with site code C16.0, which presented the cardioesophageal, esophagogastric, and gastroesophageal junctions. GNCC was classified with site codes C16.1–C16.9.

### 2.3 Statistical analysis

The distribution of clinical and pathological data was examined overall and by age groups (<55, 55–64, and ≥65 years). Categorical variables were expressed by number and (percentage per group), and the difference was assessed with the *χ*^2^ test. We further analyzed the trends of histological subtypes, subsites, and comorbidity patterns using the joinpoint regression analysis. The joinpoint regression analysis was performed using Open Source software version 4.9.1.0. The independent variable was the year. The inflection point of time grouping was selected according to the distribution of data. The dependent variables were percentages per year for cancer stages, histological subtypes, subsites, and comorbidity patterns. The maximum number of joinpoints was predefined based on the number of data points (10). Linear segmented regression analysis was utilized for the model, and log transformation of the data was performed to determine the annual percentage change in the slope along with 95% CI.

To assess the robustness of the results, sensitivity analyses were conducted. We excluded patients with unclear or missing stage information. Furthermore, sensitivity analysis was performed to explore whether trends of stage change varied by age (<55, 55–64, and ≥65 years) and sex.

Analyses were performed using R (version 4.1.1) and GraphPad Prism 9 software. All statistical tests were two-sided, with significance defined as *P <*0.05.

## 3 Results

### 3.1 Clinical characteristics of EC and GC patients

There were 10,083 EC patients and 14,244 GC patients during 2010–2019. **Tables 1**, **2** show the clinical characteristics of EC and GC patients. Cancer patients were mainly men [EC: 8,432 (83.6%); GC: 10,455 (73.4%)], diagnosed at the age group of 55-64 years [EC: 4,154 (41.2%); GC: 5,245 (36.8%)], and diagnosed at stages III (EC: 3,597 [35.7%]; GC: 4,180 [29.3%]). The most common comorbidity was hypertension [EC: 2,714 (26.9%); GC: 2,939 (20.6%)] (**Table S1**). For EC patients, the middle third of the esophagus was the most common site [3,429 (34.0%)], and the most common histological subtype was ESCC [9,392 (93.1%)]. For GC patients, cardia was the most common site accounting for 28.9% of the 14,244 cases followed by the gastric antrum [3,737 (26.2%)]. Gastric adenocarcinoma (GA) was the most common histological subtype [12,381 (86.9%)].

**Table 1:** Demographics and clinicopathological characteristics of esophageal cancer patients.

Characteristic	Total		15-54 years old		55-64 years old		65-94 years old		*χ*^2^ value	*P*-value
	*n*	%	*n*	%	*n*	%	*N*	%		
Sex										
Male	8,432	83.6	2,100	91.2	3,576	86.1	2,756	76.0	268.199	<0.001
Female	1,651	16.4	203	8.8	578	13.9	870	24.0		
Subsites										
Upper third of the esophagus	1,108	11.0	264	11.5	496	11.9	348	9.6	39.754	<0.001
Middle third of the esophagus	3,429	34.0	739	32.1	1,409	33.9	1,281	35.3		
Lower third of the esophagus	2,248	22.3	543	23.6	887	21.4	818	22.6		
Overlapping lesion of the esophagus	929	9.2	245	10.6	405	9.7	279	7.7		
Esophagus, unspecified	2,369	23.5	512	22.2	957	23	900	24.8		
Histologic subtypes										
Carcinoma, NOS	203	2.0	40	1.7	69	1.7	94	2.6	23.019	0.011
Small cell carcinoma, NOS	123	1.2	30	1.3	37	0.9	56	1.5		
Squamous cell carcinoma, NOS	9,392	93.1	2,166	94.1	3,893	93.7	3,333	91.9		
Lymphoepithelial carcinoma	99	1.0	18	0.8	44	1.1	37	1.0		
Adenocarcinoma, NOS	107	1.1	20	0.9	41	1.0	46	1.3		
Other	159	1.6	29	1.3	70	1.7	60	1.7		
Stage										
I	1,457	14.5	303	13.2	641	15.4	513	14.1	34.082	<0.001
II	2,091	20.7	434	18.8	872	21.0	785	21.6		
III	3,597	35.7	814	35.3	1,437	34.6	1,346	37.1		
IV	1,074	10.7	289	12.5	451	10.9	334	9.2		
Missing or unclear	1,864	18.5	463	20.1	753	18.1	648	17.8		
Year of diagnosis										
2010	912	9.0	241	10.5	373	9.0	298	8.2	85.269	<0.001
2011	951	9.4	244	10.6	386	9.3	321	8.9		
2012	895	8.9	242	10.5	391	9.4	262	7.2		
2013	909	9.0	199	8.6	410	9.9	300	8.3		
2014	989	9.8	237	10.3	389	9.4	363	10.0		
2015	1,086	10.8	265	11.5	447	10.7	374	10.3		
2016	1,045	10.4	235	10.2	430	10.3	380	10.5		
2017	1,068	10.6	231	10.0	428	10.3	409	11.3		
2018	1,004	10.0	203	8.8	402	9.7	399	11.0		
2019	1,224	12.1	206	9.0	498	12.0	520	14.3		
Total	10,083	100.0	2303	22.8	4154	41.2	3,626	36.0		

**Table 2:** Demographics and clinicopathological characteristics of gastric cancer patients.

Characteristic	Total		17-54 years old		55-64 years old		65-94 years old		*χ*^2^ value	*P*-value
	*n*	*%*	*n*	%	*n*	%	*n*	%		
Sex										
Male	10,455	73.4	2,936	65.1	4,037	77.0	3,482	77.6	234.188	<0.001
Female	3,789	26.6	1,575	34.9	1,208	23.0	1,006	22.4		
Subsites										
Cardia	4,112	28.9	760	16.8	1,614	30.8	1,738	38.7	582.866	<0.001
Fundus	181	1.3	50	1.1	73	1.4	58	1.3		
Body	1503	10.6	642	14.2	514	9.8	347	7.7		
Gastric antrum	3,737	26.2	1,341	29.7	1,377	26.3	1,019	22.7		
Pylorus	28	0.2	9	0.2	12	0.2	7	0.2		
Lesser curvature	508	3.6	174	3.9	188	3.6	146	3.3		
Greater curvature	106	0.7	41	0.9	43	0.8	22	0.5		
Overlapping lesion	1,071	7.5	402	8.9	386	7.4	283	6.3		
Gastric cancer, NOS	2,998	21.0	1,092	24.2	1,038	19.8	868	19.3		
Histologic subtypes										
Carcinoma, NOS	301	2.1	95	2.1	110	2.1	96	2.1	151.991	<0.001
Adenocarcinoma, NOS	12,381	86.9	3,829	84.9	4,576	87.2	3,976	88.6		
Carcinoid tumor, malignant	205	1.4	70	1.6	84	1.6	51	1.1		
Mucinous adenocarcinoma	311	2.2	74	1.6	128	2.4	109	2.4		
Signet ring cell carcinoma	467	3.3	256	5.7	137	2.6	74	1.6		
Stromal sarcoma	298	2.1	116	2.6	99	1.9	83	1.8		
Other	281	2.0	71	1.6	111	2.1	99	2.2		
Stage										
I	2,559	18.0	849	18.8	975	18.6	735	16.4	93.816	<0.001
II	2,079	14.6	548	12.1	795	15.2	736	16.4		
III	4,180	29.3	1,242	27.5	1,540	29.4	1,398	31.1		
IV	1,886	13.2	730	16.2	642	12.2	514	11.5		
Missing or unclear	3,540	24.8	1,142	25.3	1,293	24.7	1,105	24.6		
Year of diagnosis										
2010	1,205	8.4	436	9.7	431	8.2	338	7.5	59.144	<0.001
2011	1,315	9.2	454	10.1	481	9.2	380	8.5		
2012	1,288	9.0	423	9.4	481	9.2	384	8.6		
2013	1,419	10.0	484	10.7	535	10.2	400	8.9		
2014	1,447	10.2	420	9.3	536	10.2	491	10.9		
2015	1,439	10.1	470	10.4	509	9.7	460	10.2		
2016	1,378	9.7	428	9.5	515	9.8	435	9.7		
2017	1,496	10.5	462	10.2	555	10.6	479	10.7		
2018	1,526	10.7	456	10.1	572	10.9	498	11.1		
2019	1,731	12.2	478	10.6	630	12.0	623	13.9		
Total	14,244	100.0	4,511	31.7	5,245	36.8	4,488	31.5		

### 3.2 Changing trajectories of sex and age in EC and GC patients

As shown in **Tables 3**, **4**, we found an overall stable trend in the distributions of sex of EC and GC patients from 2010 to 2019. An increasing trend of percentages of patients over 65 years old was observed for EC [average annual percent change (AAPC), 3.2%; 95% CI, 1.9% to 4.5%] and GC (AAPC, 2.3%; 95% CI, 1.0% to 3.6%).

**Table 3:** Joinpoint average percent change (APC), average annual percent change (AAPC), and 95% confidence intervals (CI) for the clinical characteristics of esophageal cancer patients, 2010–2019.

	Period^a^	APC (95% CI)	*P*-value	AAPC (95% CI)	*P*-value
Sex					
Male	2010-2015	0.9 (−0.1 to 1.9)	0.073	0.5 (−0.1 to 1.1)	0.123
	2015-2019	−0.1 (−1.3 to 1.1)	0.904		
Female	2010-2016	−3.6 (−7.3 to 0.2)	0.057	−2.2 (−5.6 to 1.4)	0.222
	2016-2019	0.8 (−10.3 to 13.3)	0.864		
Age					
17-54 years old	2010-2019	−3.7 (−5.5 to −1.9)	0.001	−3.7 (−5.5 to −1.9)	0.001
55-64 years old	2010-2019	−0.5 (−1.6 to 0.5)	0.278	−0.5 (−1.6 to 0.5)	0.278
65-94 years old	2010-2019	3.2 (1.9 to 4.5)	<0.001	3.2 (1.9 to 4.5)	<0.001
Stage					
Stage I	2010-2019	10.0 (5.0 to 15.2)	0.001	10.0 (5.0 to 15.2)	0.001
Stage II	2010-2019	−6.2 (−9.1 to −3.1)	0.002	−6.2 (−9.1 to −3.1)	0.002
Stage III	2010-2017	1.4 (−0.4 to 3.3)	0.110	−1.9 (−4.8 to 1.0)	0.191
	2017-2019	−12.7 (−25.7 to 2.5)	0.081		
Stage IV	2010-2019	5.5 (2.7 to 8.4)	0.002	5.5 (2.7 to 8.4)	0.002
Missing or unclear	2010-2019	0.1 (−3.3 to 3.7)	0.935	0.1 (−3.3 to 3.7)	0.935
Histologic subtypes					
Other	2010-2019	4.2 (0.4 to 8.1)	0.033	4.2 (0.4 to 8.1)	0.033
Carcinoma, NOS	2010-2013	−25.7 (−41.9 to −5.1)	0.026	−6.9 (−14.4 to 1.2)	0.095
	2013-2019	4.2 (−6.8 to 16.5)	0.387		
Small cell carcinoma, NOS	2010-2019	−10.7 (−17.4 to −3.5)	0.010	−10.7 (−17.4 to −3.5)	0.010
Squamous cell carcinoma, NOS	2010-2019	0.3 (−0.0 to 0.5)	0.058	0.3 (−0.0 to 0.5)	0.058
Lymphoepithelial carcinoma	2010-2019	−4.1 (−10.4 to 2.6)	0.188	−4.1 (−10.4 to 2.6)	0.188
Adenocarcinoma, NOS	2010-2014	17.1 (−6.6 to 46.7)	0.132	2.4 (−6.5 to 12.1)	0.613
	2014-2019	−8.1 (−18.1 to 3.2)	0.121		
Subsites					
Upper third of the esophagus	2010-2019	−4.7 (−8.0 to −1.2)	0.015	−4.7 (−8.0 to −1.2)	0.015
Middle third of the esophagus	2010-2019	−6.5 (−8.9 to −4.1)	<0.001	−6.5 (−8.9 to −4.1)	<0.001
Lower third of the esophagus	2010-2019	2.5 (0.0 to 5.0)	0.047	2.5 (0.0 to 5.0)	0.047
Number of comorbidities					
0	2010-2019	−2.7 (−3.5 to −1.9)	<0.001	−2.7 (−3.5 to −1.9)	<0.001
1	2010-2012	20.9 (−14.0 to 69.8)	0.211	6.2 (0.0 to 12.7)	0.049
	2012-2019	2.3 (−0.4 to 5.1)	0.078		
2	2010-2019	13.0 (8.7 to 17.4)	<0.001	13.0 (8.7 to 17.4)	<0.001
≥3	2010-2019	27.7 (14.7 to 42.2)	0.001	27.7 (14.7 to 42.2)	0.001
Types of comorbidities					
Ulcerative disease	2010-2019	−5.7 (−13.6 to 3.0)	0.165	−5.7 (−13.6 to 3.0)	0.165
Inflammatory diseases	2010-2019	10.0 (4.8 to 15.5)	0.002	10.0 (4.8 to 15.5)	0.002
Metabolic diseases	2010-2019	6.1 (4.2 to 8.1)	<0.001	6.1 (4.2 to 8.1)	<0.001
Polyp disease	2010-2019	13.8 (6.6 to 21.4)	0.002	13.8 (6.6 to 21.4)	0.002

a The inflection point of time grouping was selected according to the distribution of data.

**Table 4:** Joinpoint average percent change (APC), average annual percent change (AAPC), and 95% confidence intervals (CI) for the clinical characteristics of gastric cancer patients, 2010–2019.

	Period^a^	APC (95% CI)	*P*-value	AAPC (95% CI)	*P*-value
Sex					
Male	2010-2019	−0.0 (−0.5 to 0.4)	0.915	−0.0 (−0.5 to 0.4)	0.915
Female	2010-2019	0.2 (−1.0 to 1.4)	0.720	0.2 (−1.0 to 1.4)	0.720
Age					
17-54 years old	2010-2019	−2.4 (−3.4 to −1.3)	0.001	−2.4 (−3.4 to −1.3)	0.001
55-64 years old	2010-2019	0.1 (−0.4 to 0.6)	0.691	0.1 (−0.4 to 0.6)	0.691
65-94 years old	2010-2019	2.3 (1.0 to 3.6)	0.003	2.3 (1.0 to 3.6)	0.003
Stage					
Stage I	2010-2019	9.7 (5.0 to 14.6)	0.001	9.7 (5.0 to 14.6)	0.001
Stage II	2010-2012	−19.7 (−35.5 to −0.1)	0.050	−4.6 (−8.6 to −0.4)	0.034
	2012-2019	0.2 (−3.5 to 4.1)	0.878		
Stage III	2010-2014	13.4 (4.2 to 23.3)	0.012	4.3 (0.9 to 7.8)	0.012
	2014-2019	−2.4 (−6.1 to 1.5)	0.177		
Stage IV	2010-2019	−4.2 (−9.1 to 1.0)	0.099	−4.2 (−9.1 to 1.0)	0.099
Missing or unclear	2010-2019	−7.4 (−10.7 to −4.0)	<0.001	−7.4 (−10.7 to −4.0)	<0.001
Histologic subtypes					
Other	2010-2019	−1.4 (−5.3 to 2.6)	0.436	−1.4 (−5.3 to 2.6)	0.436
Carcinoma, NOS	2010-2015	−18.9 (−28.4 to −8.1)	0.008	−6.8 (−15.7 to 3.1)	0.173
	2015-2019	11.1 (−13.7 to 42.9)	0.333		
Adenocarcinoma, NOS	2010-2019	0.0 (−0.2 to 0.2)	0.637	0.0 (−0.2 to 0.2)	0.637
Carcinoid tumor, malignant	2010-2017	15.0 (3.1 to 28.3)	0.022	2.3 (−12.2 to 19.3)	0.770
	2017-2019	−32.1 (−70.1 to 54.4)	0.280		
Mucinous adenocarcinoma	2010-2019	−3.3 (−8.2 to 1.8)	0.166	−3.3 (−8.2 to 1.8)	0.166
Signet ring cell carcinoma	2010-2019	0.2 (−5.2 to 5.9)	0.936	0.2 (−5.2 to 5.9)	0.936
Stromal sarcoma	2010-2019	6.2 (−0.1 to 12.9)	0.053	6.2 (−0.1 to 12.9)	0.053
Subsites					
Cardia	2010-2014	5.3 (−1.1 to 12.2)	0.089	−1.6 (−4.4 to 1.3)	0.279
	2014-2019	−6.8 (−11.1 to −2.3)	0.012		
Non-cardia	2010-2014	−2.3 (−4.6 to 0.1)	0.054	0.6 (−0.4 to 1.7)	0.213
	2014-2019	3.1 (1.6 to 4.5)	0.003		
Fundus of the stomach	2010-2019	1.1 (−5.2 to 7.8)	0.708	1.1 (−5.2 to 7.8)	0.708
Body of the stomach	2010-2019	0.4 (−2.3 to 3.1)	0.768	0.4 (−2.3 to 3.1)	0.768
Gastric antrum	2010-2017	2.3 (0.7 to 4.0)	0.015	−0.8 (−3.0 to 1.5)	0.489
	2017-2019	−10.9 (−21.0 to 0.3)	0.054		
Number of comorbidities					
0	2010-2019	−3.7 (−5.1 to −2.4)	<0.001	−3.7 (−5.1 to −2.4)	<0.001
1	2010-2013	11.1 (3.7 to 19.4)	0.011	4.4 (2.4 to 6.5)	<0.001
	2013-2019	1.2 (−0.4 to 2.8)	0.121		
2	2010-2019	12.7 (8.1 to 17.5)	<0.001	12.7 (8.1 to 17.5)	<0.001
≥3	2010-2019	9.3 (−25.6 to 60.6)	0.609	9.3 (−25.6 to 60.6)	0.609
Types of comorbidities					
Ulcerative disease	2010-2019	−6.1 (−11.6 to −0.3)	0.041	−6.1 (−11.6 to −0.3)	0.041
Inflammatory diseases	2010-2016	62.1 (34.8 to 95.0)	0.001	18.9 (−10.2 to 57.3)	0.193
	2016-2019	10.1 (0.1 to 21.2)	0.048		
Metabolic diseases	2010-2013	13.4 (2.2 to 25.8)	0.027	5.5 (2.5 to 8.6)	<0.001
	2013-2019	1.8 (−0.4 to 4.1)	0.095		
Polyp disease	2010-2019	7.4 (−0.4 to 15.7)	0.060	7.4 (−0.4 to 15.7)	0.060

a The inflection point of time grouping was selected according to the distribution of data.

### 3.3 Changing trajectories of stage in EC and GC patients

**Figure 1:**
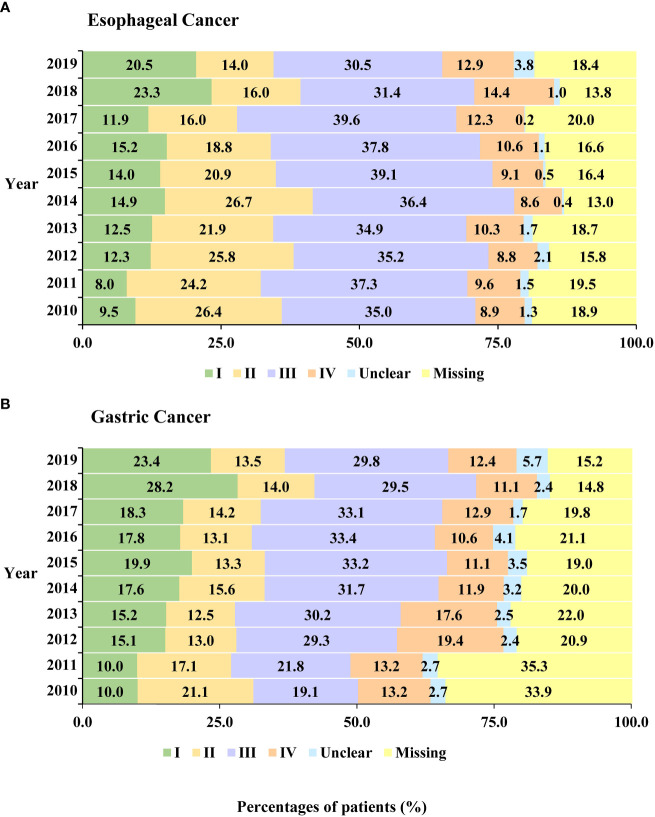
Stage distribution for all cancers from 2010 to 2019: **(A)** esophageal cancer and **(B)** gastric cancer.

For EC patients, stage-specific comparisons revealed significant increases in the percentages of stage I or stage IV (AAPC for stage I, 10.5%; 95% CI, 5.0% to 15.2%; stage IV, 5.5%; 95% CI, 2.7% to 8.4%), while the percentages of stage II decreased from 2010 to 2019 (AAPC, −6.2%; 95% CI, −9.1% to −3.1%) (**Table 3**, **Figure 1A**). The identified stage-changing trajectories significantly varied by age and sex (**Tables S2, S3**). We found that the percentages of stage IV showed significant increases in men and in the age group of 55-64 years.

For GC patients, there were significant increases in the percentages of stage I and III cases from 2010 to 2019 (AAPC for stage I, 9.7%; 95% CI, 5.0% to 14.6%; stage III, 4.3%; 95% CI, 0.9% to 7.8%). However, we observed a decreasing trend of percentages in GC patients diagnosed at stage II (AAPC, −4.6%; 95% CI, −8.6% to −0.4%) (**Table 4**, **Figure 1B**). Further analyses on the trends of age- and sex-specific percentages of stage for GC patients are shown in **Tables S2, S3**. Significant increasing trends were found in women and under the age of 55 with stage III.

The results of the sensitivity analysis were similar to those of the main analysis (**Figure S1**, **Tables S4–S7**). But we also observed significantly decreased trends of percentages in GC patients with stage IV by age (<55, 55–64, and ≥65 years) and sex.

### 3.4 Changing trajectories of comorbidity patterns in EC and GC patients

There has been an increase in EC patients with comorbidities from 2010 to 2019 (**Figures S2A, B**), and the patients with three or more comorbidities increased from 0.1% to 2.2% (AAPC, 27.7%; 95% CI, 14.7% to 42.2%) (**Table 3**). Further analyses on the trends of comorbidity patterns are shown in **Table 3**. The inflammatory comorbidity rate for EC patients increased from 1.6% in 2010 to 6.7% in 2019 (AAPC, 10.0%; 95% CI, 4.8% to 15.5%). The metabolic comorbidity rate increased from 18.0% to 35.9%, with a marked increase since 2010 (AAPC, 6.1%; 95% CI, 4.2% to 8.1%). The polyp comorbidity rate increased from 0.2% in 2010 to 1.4% in 2019 (AAPC, 13.8%; 95% CI, 6.6% to 21.4%). Metabolic and inflammatory comorbidities showed the same trends in different age groups (**Table S8**).

The percentages of GC patients with comorbidities also showed an increasing trend (**Figures S2, D**), and the trends of patients with one or two comorbidities had statistical significance (AAPC for one comorbidity, 4.4%; 95% CI, 2.4% to 6.5%; two comorbidities, 12.7%; 95% CI, 8.1% to 17.5%). For comorbidity patterns, the metabolic comorbidity rate increased from 18.8% to 30.1% (AAPC, 5.5%; 95% CI, 2.5% to 8.6%). The ulcerative comorbidity rate decreased from 2.0% to 1.2% (AAPC, −6.1%; 95% CI, −11.6% to −0.3%) (**Table 4**). Ulcerative comorbidity showed different trends in age groups, with the rate decreasing in patients over 65 years old but not in other age groups (**Table S8**).

### 3.5 Changing trajectories of histological subtypes and subsites in EC patients

**Figure 2:**
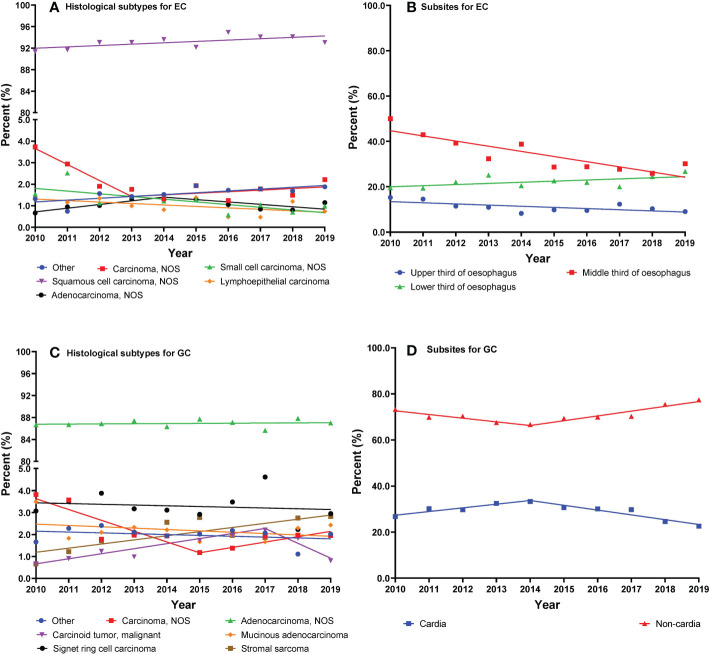
**(A–D)** Trajectories of cancer histological subtypes or subsites (lines are modeled by segmental line regression and points represent actual data).

The trends of EC cancer histological subtypes and subsites are shown in **Figures 2A, B**. We found overall stable trends of the distributions of ESCC (AAPC, 0.3%; 95% CI, −0.0% to 0.5%) and EAC (AAPC, 2.4%; 95% CI, −6.5% to 12.1%) from 2010 to 2019 (**Table 3**). The trends of histological subtypes varied by age (**Table S9**). We found a slight upward trend of percentages in ESCC patients over 65 years old. In addition, we investigated the trends of EC cancer histological subtypes by stage and found that the percentages of ESCC showed an increase in stage I and stage IV (**Table S10**).

As shown in **Table 3**, we observed a decreasing trend of the middle third of the esophagus from 2010 to 2019 (AAPC, −6.5%; 95% CI, −8.9% to −4.1%). Moreover, the percentages of the lower third of the esophagus showed an increasing trend (AAPC, 2.5%; 95% CI, 0.0% to 5.0%). We investigated the trends of EC subsites by category of sex (**Table S11**). The percentage of the middle third of the esophagus decreased in both men and women. However, the percentages of the lower third of the esophagus increased in men and remained stable in women.

### 3.6 Changing trajectories of histological subtypes and subsites in GC patients

The trends of GC cancer histological subtypes and subsites are shown in **Figures 2C, D**. The percentages of GA showed a stable trend from 2010 to 2019 (AAPC, 0.0%; 95% CI, −0.2% to 0.2%) (**Table 4**). We present the trends of histological subtypes by category of age groups (**Table S8**). GA showed a significant decrease in younger patients from 2010 to 2017 and a stable trend from 2017 to 2019. Conversely, a significant increase of GA in elderly patients was observed from 2010 to 2016, after which the percentages remained stable from 2016 to 2019. We observed an increasing trend of gastric stromal sarcoma in elderly patients from 2010 to 2019. In addition, we investigated the trends of GC cancer histological subtypes by stage and found that the percentages of GA showed an increase in stage I but a decrease in stage IV (**Table S12**).

As shown in **Table 4**, we observed that the percentages of GCC decreased from 33.3% in 2014 to 22.6% in 2019 (APC, −6.8%; 95% CI, −11.1% to −2.3%). We investigated the changing trends of subsites of GC by sex (**Table S11**). The percentages of GCC have significantly decreased in men from 2014 to 2019 and in women from 2012 to 2019.

## 4 Discussion

In this analysis, we found that the majority of EC and GC patients were men and were diagnosed at 55-64 years old. An increasing trend of percentages of patients over 65 years old was observed. The most common comorbidity was metabolic comorbidity, with hypertension being predominant. Although an increasing trend in patients diagnosed at stage I was observed from 2010 to 2019, it was still lower than that of the later stage. We observed that the middle third of the esophagus was the most common site and ESCC remained as the prioritized histological subtype of EC in China. We found overall stable trends of the distributions in ESCC and EAC from 2010 to 2019, although ESCC specifically presented an increased trend in the proportion of patients diagnosed at stages I and IV. The majority of GC patients had adenocarcinoma, and cardia was the most common site.

### 4.1 Clinical characteristics of EC and GC patients

Similar to existing evidence, we found that most EC and GC patients were diagnosed at the age of 55-64 years (11). The upward trend in the age group over 65 has some reference significance for the current screening program. The guidelines in China recommended EC and GC screening in the population between the ages of 40 and 69 years, and the age limit in urban areas was expanded to 74 years old in 2016 (12). With the increase in life expectancy, the aggravation of population aging, and the changing of age composition of EC and GC patients, the screening age can be formulated according to the specific situation of the population in different regions, so as to allocate medical resources better.

We observed that EC and GC patients were mainly men, which was consistent with previous findings (1, 13–15). Stable patterns of sex differences were found in this analysis, with a ratio of approximately 5.0:1 for EC and 3.0:1 for GC. However, the male-to-female ratio for EC in our analysis was higher than the result of the study of Zeng et al. (2.9:1) (11), being attributed to the fact that we conducted our analysis involving a single center. Similar patterns of sex differences were reported with a significant male predominance of EC and GC and a stable incidence rate ratio for EC and GC from 2003 to 2012 (4).

As for the primary site, the middle third of the esophagus was the most common site of EC, consistent with the result of Hajmanoochehri et al. (16). The cardia was the most common site accounting for 28.9% of GC cases, which was in line with the results from the USA and Germany (17, 18). The Chinese guidelines for the early diagnosis and treatment of upper gastrointestinal cancer recommended taking biopsies from the abnormal parts of the esophageal mucosa, especially positive areas with iodine staining; if there are no positive areas, a mucosal biopsy is performed at any position 25 cm from the incisors. The guidelines also recommended taking biopsies from the cardia for GC. The distribution of subsites of EC and GC in this analysis was consistent with current guidelines (12). Moreover, these findings provide further evidence for the clinical diagnosis and treatment of EC and GC and that clinicians should pay more attention to these common subsites during endoscopic examination and clinical treatments.

In addition, our result explained that ESCC remained as the prioritized histological subtype of EC, which was consistent with previous findings in most of Asia and Sub-Saharan Africa (19, 20). For ESCC, the overall burden was concentrated in Asia, where more than 400,000 new cases occurred in the world. Over 50% of cases of ESCC were estimated in China alone (5). But in the USA and other Western countries, EAC is the major type of EC (19). For GC, the most common histological subtype was GA, which was consistent with international studies (21, 22). The GNCC case burden was high in Asia and also in South and Central America. The patterns for GCC were slightly different from GNCC, with the highest burden in Eastern Asia and parts of Oceania and Western Asia (5). Major risk factor differences may contribute to distinct geographic patterns (1, 23).

### 4.2 Changing trajectories of comorbidity patterns in EC and GC patients

There has been an increase in EC and GC patients with comorbidities from 2010 to 2019, which was consistent with previous findings (24, 25). In this analysis, an increasing trend of percentages of patients over 65 years old was observed for EC and GC. Previous studies have also found that the cancer incidence rate gradually increased among the older population (26). Population aging and increment in life expectancy have led to an increasing number of older patients, which may have contributed to the concurrent increase of patients with comorbidity (27). In addition, improvements in hospital registration systems may also be contributing to the increase in the number of complications. Existing studies have shown that the incidence of cancer is mainly attributable to environmental and lifestyle factors (28) and chronic infection (29). A new study suggests that the prevalence of gastrointestinal cancers is inversely associated with hypertension and diabetes (30). According to a study published in China, there was also a positive correlation between diabetes and the mortality of cancer (31). In this analysis, the most common comorbidity pattern was metabolic comorbidity, with hypertension being predominant. Previous studies also reported that metabolic comorbidity was the prioritized comorbidity pattern of EC and GC (6, 7). For EC patients, the polyp comorbidity rate increased from 2010 to 2019. Moreover, polyp comorbidity rate in EC patients increased from 2010 to 2019. Diagnostic improvements and screening programs could be responsible for this situation (25). For GC patients, the ulcerative comorbidity rate decreased from 2010 to 2019. In China, improvements in the treatment of gastric ulcers included the introduction of antibiotic treatment of pylorus in 1999 (32) and first-line ulcer therapy in 2007 (33).

### 4.3 Changing trajectories of stage in EC and GC patients

In terms of stage at diagnosis, we found that EC and GC cases were mainly diagnosed at stage III. This was also consistent with the result of Zeng et al. (11). The analysis by Zeng et al. was based on cross-sectional data, but our analysis described changing trajectories in stages based on longitudinal data. Stage-specific comparisons revealed significant increases in the percentages of EC patients diagnosed at stages I and IV from 2010 to 2019. Recent results from the Surveillance, Epidemiology, and End Results (SEER) data also showed an upward trend of the late stage for EC patients (34). For GC patients, there were significant increases in the percentages of stage I and III cases. Previous studies have also found that the percentage of stage I in GC patients increased gradually from 1998 to 2018 (35). The Chinese government has launched some screening programs. The endoscopic screening program for upper gastrointestinal cancer showed that a one-time endoscopic screening program was associated with a significant decrease in upper gastrointestinal cancer incidence and mortality (2). Another cancer screening program in urban areas covered 18 provinces and cities, including Beijing, where this study was conducted (36). Previous studies showed that early-stage cancer cases accounted for a higher proportion after endoscopy screening (37). The patients in this study came from Beijing, which is one of the urban cancer screening coverage areas, and patients across China were enrolled. Moreover, we did observe an increasing trend in the percentages of stage I in our analyses, which may be the result of a combination of factors, including screening and changes in risk factors.

### 4.4 Changing trajectories of histological subtypes and subsites in EC patients

We analyzed many details about the trends of the percentages of EC and found that ESCC and EAC showed stable trends from 2010 to 2019. A hospital-based analysis of EAC in Japan showed conflicting results. An increase in the percentages of EAC was reported from 2007 to 2014 (20). In this analysis, the percentages of ESCC showed a slight upward trend in patients over 65 years old from 2010 to 2019. According to the present Chinese guidelines on upper gastrointestinal cancer screening and surveillance, the target population is 40-69 years old (12). From this, the elderly population may need to be aware of cancer screening and diagnosis. Although the percentages of ESCC increased in the stage I and IV groups in this analysis, the most significant increase was in stage I, which may be due to the rising awareness of early treatment. We observed that EC of the middle third of the esophagus decreased from 2010 to 2019, and the percentages of the middle third of the esophagus have significantly decreased in both men and women. In a previous study, the percentages of the middle third of the esophagus showed an upward trend from 2006 to 2008 in high-risk areas of upper gastrointestinal cancer in China (38). The changes in the percentages of high and non-high incidence areas of EC may be different and need to be further explored.

### 4.5 Changing trajectories of histological subtypes and subsites in GC patients

We observed that the percentages of GCC decreased from 33.3% in 2014 to 22.6% in 2019. A hospital-based study in Japan had consistently reported that there was a decrease in the percentages of GCC from 2007 to 2015 (14). The percentages of GCC decreased from 2014 to 2019 in a hospital-based study, which may be due to treatment of a major environmental risk factor, such as the treatment of *Helicobacter pylori*. A previous study showed that *H. pylori* infection was a strong risk factor not only for GNCC but also for CGC in Chinese adults (23). The decreasing trend in GCC in this analysis contrasts with the results from Western countries, which may be due to GCC having a different etiologic background from different countries. Most studies in Europe, the USA, and Australia have reported either null or reduced risks of GCC associated with *H. pylori* infection (39, 40).

In this analysis, the percentages of gastric stromal sarcoma showed a significant upward trend in patients over 65 years old from 2010 to 2019. A previous study suggested that gastric stromal sarcoma is common in middle-aged and elderly men (41). Gastric stromal sarcoma is the most common gastrointestinal stromal tumor (GIST) (42). No environmental risk factors for gastric stromal sarcoma are currently known. Familial predisposition due to germline mutations of KIT or PDGFRA is the most common but a very rare known risk factor (42, 43). Future basic and experimental studies are required to help elucidate the underlying etiological mechanisms of gastric stromal sarcoma.

There are a few strengths associated with our analysis. We investigated the distributions of the histological subsites of EC and GC in China with over 24,000 cancer cases across 10 years. Secondly, the accurate and detailed information collected in this study enabled us to evaluate the 10-year changing trajectories for EC and GC overall and by sex, age group, histological subtypes, or subsites. Our study has several limitations. First, this is a single-center hospital-based study, which limited its generalizability to other populations. Further study is needed to include more hospitals covering different geographic regions and areas with various economic development levels. Secondly, the time span of this study is somewhat short, and some meaningful results may not have been obtained. Thirdly, we did not collect information on personal habits and survival outcomes; therefore, we were unable to evaluate these variables in our analysis.

In conclusion, we described the clinical characteristics and trends of esophageal and gastric cancer based on 10 years of longitudinal data. According to our results, EC and GC patients were mainly men and diagnosed in the age group of 55-64 years. Metabolic comorbidity predominates in EC and GC patients. Although we observed an increasing trend of the percentages of patients diagnosed at stage I from 2010 to 2019, most patients were still diagnosed at the late stage. The middle third of the esophagus and the cardia were the most common sites of EC and GC, respectively. Moreover, changing trajectories of comorbidity patterns could have influenced the decision to operate a patient. These findings provide scientific evidence to help us identify the high-risk population for cancer screening and guide future clinical diagnosis and treatment for EC and GC.

## Data availability statement

The datasets presented in this article are not readily available because we collected the clinical and pathological data of inpatients with EC or GC from a large cancer hospital. These data cannot be shared without permission. Requests to access the datasets should be directed to wangshaoming@cicams.ac.cn.

## Author contributions

SMW and YL gave substantial contributions to the conception or the design of the manuscript. QW and XZ were responsible for the data analysis and wrote the original draft. YZ and SJW revised the manuscript draft critically. LL and WW were responsible for the visualization. FL completed the data curation. SMW provided funding support. All authors contributed to the article and approved the submitted version.
