# Supplementary material for: Clinical characteristics and changing trajectories of esophageal cancer and gastric cancer in China from 2010 to 2019: An analysis of a hospital-based database of 24,327 patients
Source: Front Oncol. 2023 Mar 13;13:1126841. doi: 10.3389/fonc.2023.1126841 (PMC10040833; doi:10.3389/fonc.2023.1126841)
Supplement: Supplementary file 1 [file DataSheet_1.docx]

Supplementary Material

Clinical characteristics and changing trajectories of esophageal cancer and gastric cancer in China from 2010 to 2019: an analysis of a hospital-based database of 24 327 patients

Wang Qiang1†, Zhang Xiaorui2†, Zhong Yuxin3, Wei Shijing1, Li Li4, Wei Wenqiang5, Liu Fen2, Li Yong6,7*, Wang Shaoming5*

*** Correspondence:**

Shaoming Wang: [wangshaoming@cicams.ac.cn](mailto:wangshaoming@cicams.ac.cn); Yong Li: liyongdoctor@126.com

# Supplementary Figures and Tables

## Supplementary Figures


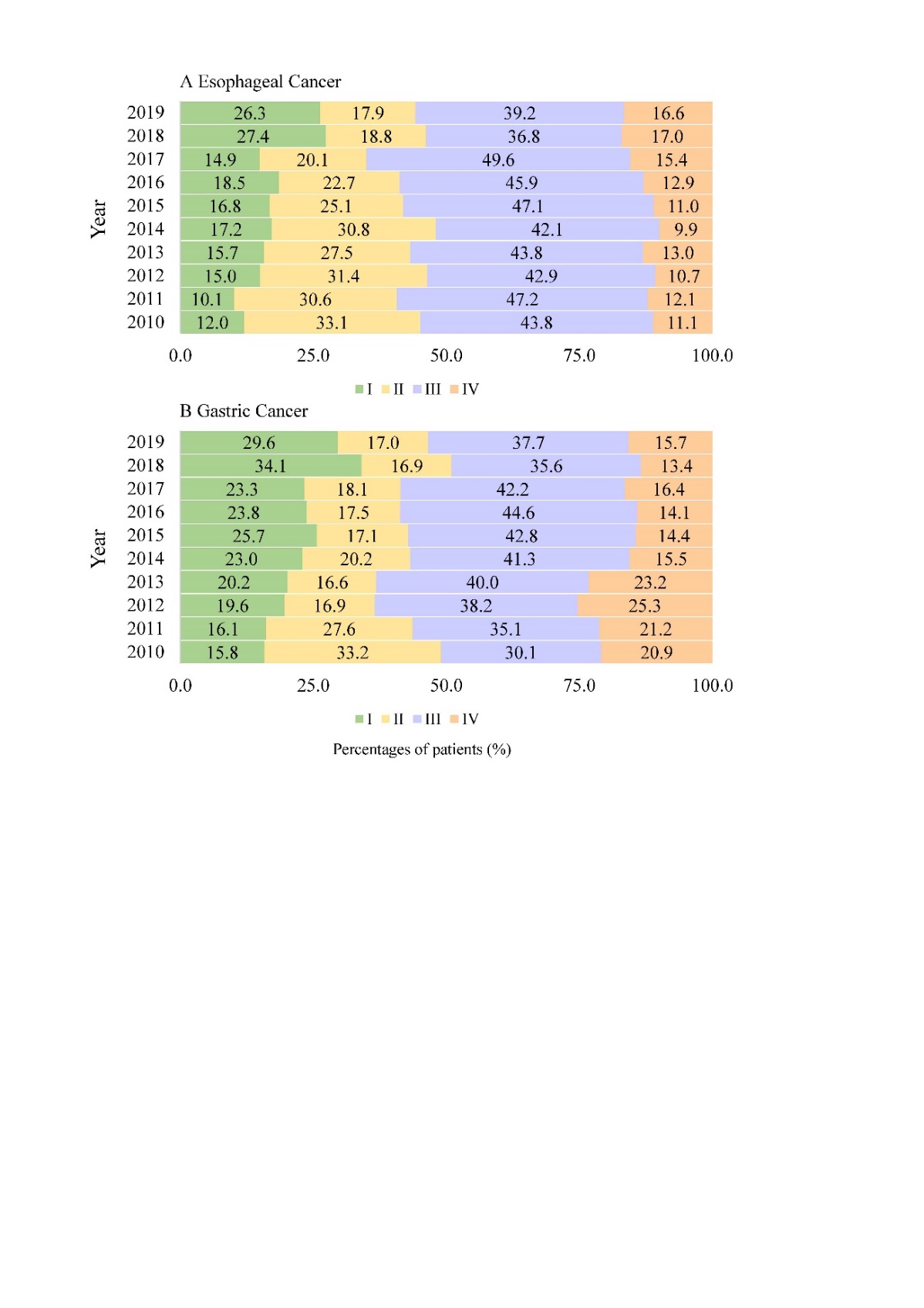


**Supplementary Figure 1.** Stage distribution for all cancers from 2010 to 2019 in sensitivity analysis (A: Esophageal cancer; B: Gastric cancer).


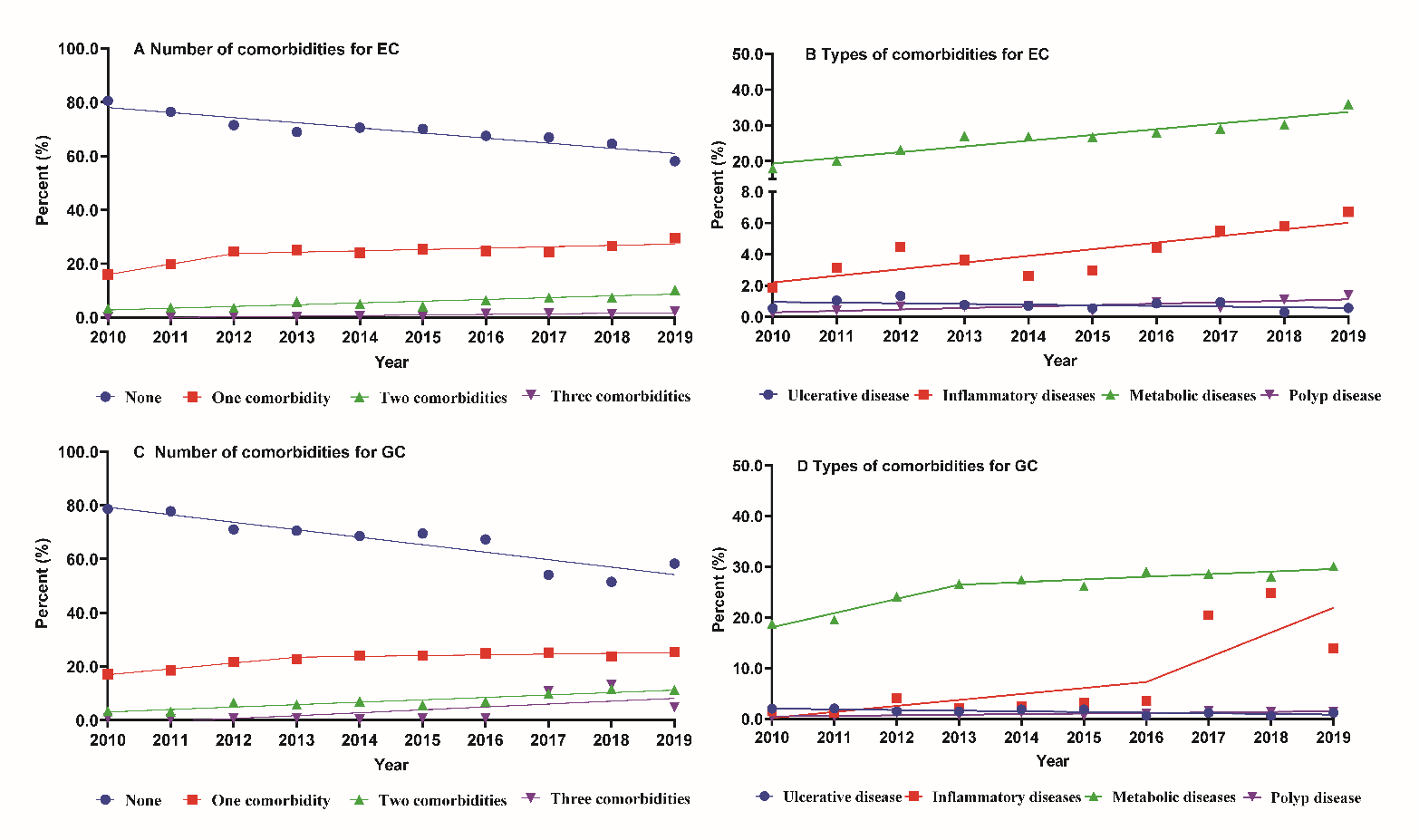


**Supplementary Figure 2.** Trajectories of comorbidities for esophageal cancer and gastric cancer (Lines are modeled by segmental line regression and points represent actual data.)

## Supplementary Tables

**Supplementary Table 1** Comorbidity patterns of esophageal cancer and gastric cancer.

|  | Esophageal cancer | |  | Gastric cancer | |
| --- | --- | --- | --- | --- | --- |
| Comorbidity patterns | *n* | *%* |  | *n* | *%* |
| **Ulcerative comorbidities** | 76 | 0.8 |  | 201 | 1.4 |
| Duodenal | 29 | 0.3 |  | 62 | 0.4 |
| Gastric ulcers | 50 | 0.5 |  | 143 | 1.0 |
| **Inflammatory comorbidities** | 423 | 4.2 |  | 1171 | 8.2 |
| Gastritis | 421 | 4.2 |  | 1157 | 8.1 |
| Atrophic gastritis | 109 | 1.1 |  | 629 | 4.4 |
| Ulcerative colitis | 0 | 0.0 |  | 3 | 0.0 |
| Appendicitis | 2 | 0.0 |  | 8 | 0.1 |
| **Metabolic comorbidities** | 2714 | 26.9 |  | 3725 | 26.2 |
| Hypertension | 2245 | 22.3 |  | 2939 | 20.6 |
| Diabetes | 867 | 8.6 |  | 1361 | 9.6 |
| Hyperlipemia | 35 | 0.3 |  | 61 | 0.4 |
| Hypercholesterolemia | 1 | 0.0 |  | 1 | 0.0 |
| **Polyp comorbidities** | 73 | 0.7 |  | 146 | 1.0 |
| Intestinal polyps | 34 | 0.3 |  | 73 | 0.5 |
| Gastric polyps | 40 | 0.4 |  | 75 | 0.5 |
| Esophageal polyps | 1 | 0.0 |  | 0 | 0.0 |

**Supplementary Table 2** Changing trajectories of stage for esophageal cancer and gastric cancer, by age group, 2010–2019

|  | <55 years old | | | | |  | 55-64 years old | | | | |  | ≥65 years old | | | | |
| --- | --- | --- | --- | --- | --- | --- | --- | --- | --- | --- | --- | --- | --- | --- | --- | --- | --- |
|  | Period | APC (95%CI) | *P* value | AAPC (95%CI) | *P* value |  | Period | APC (95%CI) | *P* value | AAPC (95%CI) | *P* value |  | Period | APC (95%CI) | *P* value | AAPC (95%CI) | *P* value |
| **Esophageal**  **cancer** |  |  |  |  |  |  |  |  |  |  |  |  |  |  |  |  |  |
| I | 2010-2019 | 11.9(6.9 to 17.3) | 0.001 | 11.9(6.9 to 17.3) | 0.001 |  | 2010-2019 | 10.9(4.6 to 17.5) | 0.003 | 10.9(4.6 to 17.5) | 0.003 |  | 2010-2019 | 7.2(2.7 to 11.8) | 0.006 | 7.2(2.7 to 11.8) | 0.006 |
| II | 2010-2019 | -5.3(-10.8 to 0.5) | 0.066 | -5.3(-10.8 to 0.5) | 0.066 |  | 2010-2019 | -8.2(-10.3 to -6.0) | <0.001 | -8.2(-10.3 to -6.0) | <0.001 |  | 2010-2014 | 3.4(-5.7 to 13.4) | 0.394 | -4.8(-8.8 to -0.6) | 0.026 |
|  |  |  |  |  |  |  |  |  |  |  |  |  | 2014-2019 | -10.8(-16.8 to -4.4) | 0.008 |  |  |
| III | 2010-2019 | -0.6(-4.4 to 3.3) | 0.725 | -0.6(-4.4 to 3.3) | 0.725 |  | 2010-2017 | 2.4(0.4 to 4.5) | 0.028 | -2.0(-5.1 to 1.2) | 0.215 |  | 2010-2019 | -0.9(-3.0 to 1.2) | 0.340 | -0.9(-3.0 to 1.2) | 0.340 |
|  |  |  |  |  |  |  | 2017-2019 | -16.1(-29.7 to 0.2) | 0.052 |  |  |  |  |  |  |  |  |
| IV | 2010-2019 | 3.9(-0.4 to 8.4) | 0.071 | 3.9(-0.4 to 8.4) | 0.071 |  | 2010-2015 | 0.7(-4.6 to 6.3) | 0.751 | 5.7(2.7 to 8.7) | <0.001 |  | 2010-2019 | 5.6(-0.5 to 12.0) | 0.068 | 5.6(-0.5 to 12.0) | 0.068 |
|  |  |  |  |  |  |  | 2015-2019 | 12.2(7.0 to 17.7) | 0.002 |  |  |  |  |  |  |  |  |
| Missing or unclear | 2010-2019 | -1.4(-5.2 to 2.6) | 0.449 | -1.4(-5.2 to 2.6) | 0.449 |  | 2010-2019 | 0.5(-2.9 to 4.1) | 0.737 | 0.5(-2.9 to 4.1) | 0.737 |  | 2010-2019 | 0.9(-3.7 to 5.8) | 0.659 | 0.9(-3.7 to 5.8) | 0.659 |
| **Gastric**  **cance**r |  |  |  |  |  |  |  |  |  |  |  |  |  |  |  |  |  |
| I | 2010-2019 | 8.2(3.8 to 12.9) | 0.002 | 8.2(3.8 to 12.9) | 0.002 |  | 2010-2019 | 10.5(5.0 to 16.3) | 0.002 | 10.5(5.0 to 16.3) | 0.002 |  | 2010-2019 | 10.2(4.5 to 16.2) | 0.003 | 10.2(4.5 to 16.2) | 0.003 |
| II | 2010-2019 | -2.6(-6.1 to 1.0) | 0.128 | -2.6(-6.1 to 1.0) | 0.128 |  | 2010-2012 | -22.5(-39.6 to -0.4) | 0.048 | -5.5(-10.3 to -0.6) | 0.029 |  | 2010-2012 | -21.8(-36.5 to -3.7) | 0.029 | -5.3(-9.2 to -1.3) | 0.010 |
|  |  |  |  |  |  |  | 2012-2019 | -0.1(-4.8 to 4.9) | 0.973 |  |  |  | 2012-2019 | 0.0(-3.7 to 3.9) | 0.989 |  |  |
| III | 2010-2013 | 21.6(1.0 to 46.4) | 0.042 | 7.2(1.8 to 12.8) | 0.008 |  | 2010-2019 | 2.3(-1.5 to 6.2) | 0.201 | 2.3(-1.5 to 6.2) | 0.201 |  | 2010-2013 | 19.4(-1.2 to 44.3) | 0.061 | 4.1(-1.3 to 9.7) | 0.136 |
|  | 2013-2019 | 0.6(-3.2 to 4.5) | 0.701 |  |  |  |  |  |  |  |  |  | 2013-2019 | -2.9(-6.7 to 1.2) | 0.129 |  |  |
| IV | 2010-2019 | -4.1(-8.9 to 1.0) | 0.099 | -4.1(-8.9 to 1.0) | 0.099 |  | 2010-2019 | -3.4(-8.5 to 2.0) | 0.178 | -3.4(-8.5 to 2.0) | 0.178 |  | 2010-2019 | -4.7(-10.4 to 1.4) | 0.113 | -4.7(-10.4 to 1.4) | 0.113 |
| Missing or unclear | 2010-2019 | -8.6(-12.2 to -5.0) | 0.001 | -8.6(-12.2 to -5.0) | 0.001 |  | 2010-2019 | -8.2(-11.6 to -4.6) | 0.001 | -8.2(-11.6 to -4.6) | 0.001 |  | 2010-2019 | -4.9(-8.5 to -1.3) | 0.014 | -4.9(-8.5 to -1.3) | 0.014 |

**Supplementary Table 3** Changing trajectories of stage for esophageal cancer and gastric cancer, by sex group, 2010–2019

|  | Male | | | | |  | Female | | | | |
| --- | --- | --- | --- | --- | --- | --- | --- | --- | --- | --- | --- |
|  | Period | APC (95%CI) | *P* value | AAPC (95%CI) | *P* value |  | Period | APC (95%CI) | *P* value | AAPC (95%CI) | *P* value |
| **Esophageal cancer** |  |  |  |  |  |  |  |  |  |  |  |
| Stage I | 2010-2019 | 11.0(5.8 to 16.4) | 0.001 | 11.0(5.8 to 16.4) | 0.001 |  | 2010-2019 | 6.2(0.2 to 12.5) | 0.043 | 6.2(0.2 to 12.5) | 0.043 |
| Stage II | 2010-2019 | -6.1(-9.6 to -2.6) | 0.005 | -6.1(-9.6 to -2.6) | 0.005 |  | 2010-2019 | -5.6(-8.6 to -2.5) | 0.004 | -5.6(-8.6 to -2.5) | 0.004 |
| Stage III | 2010-2017 | 0.9(-0.6 to 2.4) | 0.184 | -2.4(-4.7 to -0.0) | 0.048 |  | 2010-2019 | 1.7(-1.9 to 5.4) | 0.307 | 1.7(-1.9 to 5.4) | 0.307 |
|  | 2017-2019 | -13.2(-23.9 to -0.9) | 0.041 |  |  |  |  |  |  |  |  |
| Stage IV | 2010-2019 | 5.6(3.4 to 7.9) | <0.001 | 5.6(3.4 to 7.9) | <0.001 |  | 2010-2019 | 4.1(-3.1 to 11.8) | 0.232 | 4.1(-3.1 to 11.8) | 0.232 |
| Missing or unclear | 2010-2019 | 0.2(-3.5 to 4.1) | 0.886 | 0.2(-3.5 to 4.1) | 0.886 |  | 2010-2019 | -1.0(-4.2 to 2.2) | 0.481 | -1.0(-4.2 to 2.2) | 0.481 |
| **Gastric cance**r |  |  |  |  |  |  |  |  |  |  |  |
| Stage I | 2010-2019 | 9.0(4.8 to 13.4) | 0.001 | 9.0(4.8 to 13.4) | 0.001 |  | 2010-2019 | 11.1(4.9 to 17.7) | 0.003 | 11.1(4.9 to 17.7) | 0.003 |
| Stage II | 2010-2012 | -17.8(-33.1 to 0.9) | 0.058 | -4.2(-8.0 to -0.2) | 0.038 |  | 2010-2012 | -24.3(-47.7 to 9.5) | 0.110 | -5.7(-12.5 to 1.7) | 0.129 |
|  | 2012-2019 | 0.1(-3.3 to 3.6) | 0.929 |  |  |  | 2012-2019 | 0.5(-6.4 to 7.8) | 0.872 |  |  |
| Stage III | 2010-2012 | 30.9(-11.0 to 92.6) | 0.133 | 6.1(-0.8 to 13.6) | 0.085 |  | 2010-2014 | 17.3(6.2 to 29.5) | 0.009 | 5.8(1.8 to 9.9) | 0.004 |
|  | 2012-2019 | -0.0(-3.0 to 3.0) | 0.975 |  |  |  | 2014-2019 | -2.6(-6.6 to 1.7) | 0.178 |  |  |
| Stage IV | 2010-2019 | -3.2(-7.7 to 1.5) | 0.151 | -3.2(-7.7 to 1.5) | 0.151 |  | 2010-2019 | -6.0(-12.1 to 0.5) | 0.066 | -6.0(-12.1 to 0.5) | 0.066 |
| Missing or unclear | 2010-2019 | -7.9(-11.3 to -4.4) | 0.001 | -7.9(-11.3 to -4.4) | 0.001 |  | 2010-2019 | -6.0(-9.6 to -2.2) | 0.007 | -6.0(-9.6 to -2.2) | 0.007 |

**Supplementary** **Table 4** Joinpoint average percent change (APC), average annual percent change (AAPC) and 95% confidence intervals (CI) of stage for esophageal cancer, 2010–2019.

|  | Period | APC (95%CI) | *P* value | AAPC (95%CI) | *P* value |
| --- | --- | --- | --- | --- | --- |
| Stage |  |  |  |  |  |
| Stage I | 2010-2019 | 10.2(6.0 to 14.7 | <0.001 | 10.2(6.0 to 14.7 | 0.001 |
| Stage II | 2010-2019 | -6.5(-8.5 to -4.3) | <0.001 | -6.5(-8.5 to -4.3) | 0.001 |
| Stage III | 2010-2019 | -0.6(-0.3 to 1.8) | 0.570 | -0.6(-0.3 to 1.8) | 0.570 |
| Stage IV | 2010-2019 | 5.4(2.5 to 8.5) | 0.003 | 5.4(2.5 to 8.5) | 0.003 |

**Supplementary Table 5** Joinpoint average percent change (APC), average annual percent change (AAPC) and 95% confidence intervals (CI) of stage for gastric cancer, 2010–2019.

|  | Period | APC (95%CI) | *P* value | AAPC (95%CI) | *P* value |
| --- | --- | --- | --- | --- | --- |
| Stage |  |  |  |  |  |
| Stage I | 2010-2019 | 7.8(4.3 to 11.4) | 0.001 | 7.8(4.3 to 11.4) | 0.001 |
| Stage II | 2010-2012 | -26.0(-38.0 to -11.7) | 0.007 | -7.4(-10.8 to -4.0) | <0.001 |
|  | 2012-2019 | -1.3(-4.8 to 2.3) | 0.381 |  |  |
| Stage III | 2010-2016 | 5.1(1.2 to 9.1) | 0.019 | 0.8(-2.0 to 3.8) | 0.568 |
|  | 2016-2019 | -7.1(-14.8 to 1.2) | 0.077 |  |  |
| Stage IV | 2010-2019 | -5.9(-9.9 to -1.8) | 0.011 | -5.9(-9.9 to -1.8) | 0.011 |

**Supplementary Table 6** Changing trajectories of stage for esophageal cancer and gastric cancer in sensitivity analysis, by age group, 2010–2019

|  | <55 years old | | | | |  | 55-64 years old | | | | |  | ≥65 years old | | | | |
| --- | --- | --- | --- | --- | --- | --- | --- | --- | --- | --- | --- | --- | --- | --- | --- | --- | --- |
|  | Period | APC (95%CI) | *P* value | AAPC (95%CI) | *P* value |  | Period | APC (95%CI) | *P* value | AAPC (95%CI) | *P* value |  | Period | APC (95%CI) | *P* value | AAPC (95%CI) | *P* value |
| **Esophageal cancer** |  |  |  |  |  |  |  |  |  |  |  |  |  |  |  |  |  |
| I | 2010-2019 | 12.3(7.7 to 17.0) | 0.001 | 12.3(7.7 to 17.0) | 0.001 |  | 2010-2019 | 11.0(5.7 to 16.6) | 0.001 | 11.0(5.7 to 16.6) | 0.001 |  | 2010-2019 | 7.5(3.1 to 12.0) | 0.004 | 7.5(3.1 to 12.0) | 0.004 |
| II | 2010-2019 | -6.0(-10.4 to -1.3) | 0.019 | -6.0(-10.4 to -1.3) | 0.019 |  | 2010-2019 | -8.2(-10.0 to -6.3) | <0.001 | -8.2(-10.0 to -6.3) | <0.001 |  | 2010-2019 | -4.7(-7.7 to -1.6) | 0.009 | -4.7(-7.7 to -1.6) | 0.009 |
| III | 2010-2019 | -0.9(-4.1 to 2.5) | 0.565 | -0.9(-4.1 to 2.5) | 0.565 |  | 2010-2017 | 2.2(0.0 to 4.4) | 0.051 | -1.9(-5.3 to 1.7) | 0.295 |  | 2010-2019 | -0.9(-3.0 to 1.4) | 0.394 | -0.9(-3.0 to 1.4) | 0.394 |
|  |  |  |  |  |  |  | 2017-2019 | -14.9(-30.1 to 3.6) | 0.088 |  |  |  |  |  |  |  |  |
| IV | 2010-2019 | 3.7(-1.3 to 9.0) | 0.125 | 3.7(-1.3 to 9.0) | 0.125 |  | 2010-2015 | 0.1(-7.7 to 8.7) | 0.996 | 5.7(1.4 to 10.3) | 0.010 |  | 2010-2019 | 5.4(-0.2 to 11.3) | 0.056 | 5.4(-0.2 to 11.3) | 0.056 |
|  |  |  |  |  |  |  | 2015-2019 | 13.1(5.5 to 21.3) | 0.006 |  |  |  |  |  |  |  |  |
| **Gastric cance**r |  |  |  |  |  |  |  |  |  |  |  |  |  |  |  |  |  |
| I | 2010-2019 | 5.8(2.2 to 9.6) | 0.006 | 5.8(2.2 to 9.6) | 0.006 |  | 2010-2019 | 8.7(4.3 to 13.2) | 0.002 | 8.7(4.3 to 13.2) | 0.002 |  | 2010-2019 | 8.8(4.3 to 13.6) | 0.002 | 8.8(4.3 to 13.6) | 0.002 |
| II | 2010-2012 | -22.4(-44.4 to 8.2) | 0.107 | -6.4(-12.3 to -0.1) | 0.046 |  | 2010-2012 | -28.1(-37.8 to -16.9) | 0.002 | -8.8(-11.6 to -5.8) | <0.001 |  | 2010-2013 | -19.9(-34.7 to -1.7) | 0.038 | -6.7(-12.5 to -0.5) | 0.034 |
|  | 2012-2019 | -1.3(-6.6 to 4.3) | 0.575 |  |  |  | 2012-2019 | -2.3(-5.6 to 1.0) | 0.131 |  |  |  | 2013-2019 | 0.7(-6.4 to 8.4) | 0.812 |  |  |
| III | 2010-2017 | 5.0(1.4 to 8.6) | 0.015 | 1.0(-3.2 to 5.4) | 0.643 |  | 2010-2019 | 0.5(-2.5 to 3.5) | 0.721 | 0.5(-2.5 to 3.5) | 0.721 |  | 2010-2015 | 6.8(-0.1 to 14.1) | 0.052 | 0.9(-2.8 to 4.7) | 0.637 |
|  | 2017-2019 | -11.7(-29.1 to 10.0) | 0.205 |  |  |  |  |  |  |  |  |  | 2015-2019 | -6.0(-12.4 to 0.9) | 0.074 |  |  |
| IV | 2010-2019 | -6.6(-10.4 to -2.6) | 0.006 | -6.6(-10.4 to -2.6) | 0.006 |  | 2010-2019 | -5.1(-9.2 to -0.8) | 0.025 | -5.1(-9.2 to -0.8) | 0.025 |  | 2010-2019 | -5.6(-10.4 to -0.5) | 0.036 | -5.6(-10.4 to -0.5) | 0.036 |

**Supplementary Table 7** Changing trajectories of stage for esophageal cancer and gastric cancer in sensitivity analysis, by sex group, 2010–2019

|  | Male | | | | |  | Female | | | | |
| --- | --- | --- | --- | --- | --- | --- | --- | --- | --- | --- | --- |
|  | Period | APC (95%CI) | *P* value | AAPC (95%CI) | *P* value |  | Period | APC (95%CI) | *P* value | AAPC (95%CI) | *P* value |
| **Esophageal cancer** |  |  |  |  |  |  |  |  |  |  |  |
| Stage I | 2010-2019 | 11.2(6.7 to 15.9) | <0.001 | 11.2(6.7 to 15.9) | <0.001 |  | 2010-2019 | 6.2(1.1 to 11.6) | 0.023 | 6.2(1.1 to 11.6) | 0.023 |
| Stage II | 2010-2019 | -6.5(-9.0 to -3.9) | 0.001 | -6.5(-9.0 to -3.9) | 0.001 |  | 2010-2019 | -5.8(-8.6 to -2.9) | 0.002 | -5.8(-8.6 to -2.9) | 0.002 |
| Stage III | 2010-2019 | -1.1(-3.2 to 1.1) | 0.294 | -1.1(-3.2 to 1.1) | 0.294 |  | 2010-2019 | 1.6(-2.2 to 5.6) | 0.353 | 1.6(-2.2 to 5.6) | 0.353 |
| Stage IV | 2010-2019 | 5.7(3.0 to 8.4) | 0.001 | 5.7(3.0 to 8.4) | 0.001 |  | 2010-2019 | 3.7(-3.1 to 11.0) | 0.252 | 3.7(-3.1 to 11.0) | 0.252 |
| **Gastric cance**r |  |  |  |  |  |  |  |  |  |  |  |
| Stage I | 2010-2019 | 7.2(4.1 to 10.5) | 0.001 | 7.2(4.1 to 10.5) | 0.001 |  | 2010-2019 | 11.1(4.9 to 17.7) | 0.003 | 11.1(4.9 to 17.7) | 0.003 |
| Stage II | 2010-2012 | -24.3(-35.4 to -11.3) | 0.006 | -7.1(-10.1 to -4.0) | <0.001 |  | 2010-2012 | -30.4(-47.5 to -7.7) | 0.021 | -8.5(-13.9 to -2.7) | 0.004 |
|  | 2012-2019 | -1.5(-4.5 to 1.6) | 0.275 |  |  |  | 2012-2019 | -1.0(-7.1 to 5.5) | 0.702 |  |  |
| Stage III | 2010-2016 | 4.2(-0.3 to 9.0) | 0.063 | 0.4(-3.0 to 4.0) | 0.802 |  | 2010-2015 | 10.8(4.9 to 17.1) | 0.005 | 2.9(-0.2 to 6.1) | 0.065 |
|  | 2016-2019 | -6.7(-15.8 to 3.3) | 0.139 |  |  |  | 2015-2019 | -6.2(-11.5 to -0.5) | 0.038 |  |  |
| Stage IV | 2010-2019 | -5.1(-8.8 to -1.3) | 0.015 | -5.1(-8.8 to -1.3) | 0.015 |  | 2010-2019 | -7.4(-12.2 to -2.5) | 0.009 | -7.4(-12.2 to -2.5) | 0.009 |

**Supplementary Table 8** Changing trajectories of comorbidity patterns for esophageal cancer and gastric cancer, by age group, 2010–2019

|  | <55 years old | | | | |  | 55-64 years old | | | | |  | ≥65 | | | | |
| --- | --- | --- | --- | --- | --- | --- | --- | --- | --- | --- | --- | --- | --- | --- | --- | --- | --- |
|  | Period | APC (95%CI) | *P* value | AAPC (95%CI) | *P* value |  | Period | APC (95%CI) | *P* value | AAPC (95%CI) | *P* value |  | Period | APC (95%CI) | *P* value | AAPC (95%CI) | *P* value |
| **Esophageal cancer** |  |  |  |  |  |  |  |  |  |  |  |  |  |  |  |  |  |
| Number of comorbidities |  |  |  |  |  |  |  |  |  |  |  |  |  |  |  |  |  |
| 0 | 2010-2019 | -1.5(-2.4 to -0.5) | 0.008 | -1.5(-2.4 to -0.5) | 0.008 |  | 2010-2012 | -6.6(-11.1 to -1.9) | 0.016 | -3.0(-4.0 to -2.0) | <0.001 |  | 2010-2019 | -2.8(-4.2 to -1.5) | 0.001 | -2.8(-4.2 to -1.5) | 0.001 |
|  |  |  |  |  |  |  | 2012-2019 | -2.0(-2.9 to -1.0) | 0.003 |  |  |  |  |  |  |  |  |
| 1 | 2010-2019 | 5.2(2.3 to 8.0) | 0.003 |  |  |  | 2010-2012 | 29.0(-17.1 to 102.0) | 0.205 | 6.5(-1.5 to 15.2) | 0.116 |  | 2010-2019 | 2.6(0.1 to 5.3) | 0.046 | 2.6(0.1 to 5.3) | 0.046 |
|  |  |  |  |  |  |  | 2012-2019 | 0.8(-2.5 to 4.2) | 0.546 |  |  |  |  |  |  |  |  |
| 2 | 2010-2017 | -3.5(-13.8 to 8.1) | 0.455 | 10.8(-3.9 to 27.8) | 0.159 |  | 2010-2019 | 15.2(9.7 to 20.9) | <0.001 | 15.2(9.7 to 20.9) | <0.001 |  | 2010-2019 | 7.0(0.5 to 13.8) | 0.037 | 7.0(0.5 to 13.8) | 0.037 |
|  | 2017-2019 | 79.6(-14.6 to 277.7) | 0.099 |  |  |  |  |  |  |  |  |  |  |  |  |  |  |
| ≥3 | - | - | - | - | - |  | - | - | - | - | - |  | - | - | - | - | - |
| Types of comorbidities |  |  |  |  |  |  |  |  |  |  |  |  |  |  |  |  |  |
| Ulcerative disease | - | - | - | - | - |  | 2010-2019 | -2.6(-12.9 to 9.0) | 0.606 | -2.6(-12.9 to 9.0) | 0.606 |  | - | - | - | - | - |
| Metabolic diseases | 2010-2019 | 3.7(0.8 to 6.8) | 0.019 | 3.7(0.8 to 6.8) | 0.019 |  | 2010-2019 | 6.0(3.4 to 8.6) | 0.001 | 6.0(3.4 to 8.6) | 0.001 |  | 2010-2019 | 5.1(2.6 to 7.7) | 0.001 | 5.1(2.6 to 7.7) | 0.001 |
| Polyp disease | - | - | - | - | - |  | 2010-2019 | 7.4(-3.9 to 19.9) | 0.177 | 7.4(-3.9 to 19.9) | 0.177 |  | - | - | - | - | - |
| Inflammatory diseases | 2010-2019 | 12.0(1.3 to 23.8) | 0.031 | 12.0(1.3 to 23.8) | 0.031 |  | 2010-2019 | 8.6(0.9 to 16.9) | 0.032 | 8.6(0.9 to 16.9) | 0.032 |  | 2010-2019 | 7.3(0.7 to 14.4) | 0.033 | 7.3(0.7 to 14.4) | 0.033 |
|  |  |  |  |  |  |  |  |  |  |  |  |  |  |  |  |  |  |
| **Gastric cance**r |  |  |  |  |  |  |  |  |  |  |  |  |  |  |  |  |  |
| Number of comorbidities |  |  |  |  |  |  |  |  |  |  |  |  |  |  |  |  |  |
| 0 | 2010-2019 | -3.0(-4.4 to -1.6) | 0.001 | -3.0(-4.4 to -1.6) | 0.001 |  | 2010-2019 | -3.8(-5.0 to -2.7) | <0.001 | -3.8(-5.0 to -2.7) | <0.001 |  | 2010-2019 | -3.7(-5.4 to -1.9) | 0.002 | -3.7(-5.4 to -1.9) | 0.002 |
| 1 | 2010-2012 | 22.7(-10.3 to 67.8) | 0.154 | 8.7(2.9 to 14.8) | 0.003 |  | 2010-2013 | 13.9(-3.9 to 35.0) | 0.106 | 3.6(-1.2 to 8.7) | 0.143 |  | 2010-2017 | 3.8(1.6 to 6.0) | 0.007 | 1.4(-1.2 to 4.0) | 0.291 |
|  | 2012-2019 | 5.0(2.6 to 7.4) | 0.003 |  |  |  | 2013-2019 | -1.1(-5.1 to 3.0) | 0.507 |  |  |  | 2017-2019 | -6.6(-18.1 to 6.6) | 0.241 |  |  |
| 2 | 2010-2019 | 21.0(7.2 to 36.5) | 0.007 | 21.0(7.2 to 36.5) | 0.007 |  | 2010-2019 | 10.7(5.8 to 15.7) | 0.001 | 10.7(5.8 to 15.7) | 0.001 |  | 2010-2019 | 8.7(3.8 to 13.9) | 0.003 | 8.7(3.8 to 13.9) | 0.003 |
| ≥3 | - | - | - | - | - |  | 2010-2019 | 8.3(-26.6 to 59.8) | 0.648 | 8.3(-26.6 to 59.8) | 0.648 |  | 2010-2019 | 22.3(-9.8 to 65.8) | 0.166 | 22.3(-9.8 to 65.8) | 0.166 |
| Types of comorbidities |  |  |  |  |  |  |  |  |  |  |  |  |  |  |  |  |  |
| Ulcerative disease | 2010-2019 | -4.9(-11.5 to 2.1) | 0.143 | -4.9(-11.5 to 2.1) | 0.143 |  | 2010-2019 | -5.8(-15.4 to 4.9) | 0.238 | -5.8(-15.4 to 4.9) | 0.238 |  | 2010-2019 | -9.4(-16.6 to -1.5) | 0.026 | -9.4(-16.6 to -1.5) | 0.026 |
| Metabolic diseases | 2010-2012 | 34.7(-8.3 to 97.9) | 0.103 | 9.5(2.4 to 17.1) | 0.008 |  | 2010-2013 | 17.7(1.6 to 36.3) | 0.036 | 5.5(1.3 to 9.9) | 0.009 |  | 2010-2019 | 2.6(1.1 to 4.1) | 0.004 | 2.6(1.1 to 4.1) | 0.004 |
|  | 2012-2019 | 3.2(0.5 to 6.0) | 0.027 |  |  |  | 2013-2019 | -0.1(-3.1 to 3.0) | 0.958 |  |  |  |  |  |  |  |  |
| Polyp disease | 2010-2019 | -0.2(-10.1 to 10.8) | 0.971 | -0.2(-10.1 to 10.8) | 0.971 |  | - | - | - | - | - |  | 2010-2019 | 5.1(-2.4 to 13.2) | 0.160 | 5.1(-2.4 to 13.2) | 0.160 |
| Inflammatory diseases | 2010-2019 | 16.2(-19.9 to 68.5) | 0.379 | 16.2(-19.9 to 68.5) | 0.379 |  | 2010-2017 | 52.1(24.6 to 85.7) | 0.003 | 34.7(19.1 to 52.4) | <0.001 |  | 2010-2019 | 21.8(-5.1 to 56.5) | 0.106 | 21.8(-5.1 to 56.5) | 0.106 |
|  |  |  |  |  |  |  | 2017-2019 | -11.8(-28.3 to 8.4) | 0.178 |  |  |  |  |  |  |  |  |

**Supplementary Table 9** Changing trajectories of histologic subtypes for esophageal cancer and gastric cancer, by age group, 2010–2019

|  | <55 years old | | | | |  | 55-64 years old | | | | |  | ≥65 | | | | |
| --- | --- | --- | --- | --- | --- | --- | --- | --- | --- | --- | --- | --- | --- | --- | --- | --- | --- |
|  | Period | APC (95%CI) | *P* value | AAPC (95%CI) | *P* value |  | Period | APC (95%CI) | *P* value | AAPC (95%CI) | *P* value |  | Period | APC (95%CI) | *P* value | AAPC (95%CI) | *P* value |
| **Esophageal cancer** |  |  |  |  |  |  |  |  |  |  |  |  |  |  |  |  |  |
| Other | 2010-2019 | 6.2(-2.7 to 16.0) | 0.152 | 6.2(-2.7 to 16.0) | 0.152 |  | 2010-2019 | 6.2(-0.5 to 13.2) | 0.065 | 6.2(-0.5 to 13.2) | 0.065 |  | 2010-2019 | -1.4(-6.2 to 3.6) | 0.523 | -1.4(-6.2 to 3.6) | 0.523 |
| Carcinoma, NOS | 2010-2019 | -8.8(-15.0 to -2.1) | 0.018 | -8.8(-15.0 to -2.1) | 0.018 |  | 2010-2016 | -22.9(-30.3 to -14.7) | 0.001 | -7.7(-18.5 to 4.4) | 0.200 |  | 2010-2019 | -3.7(-9.4 to 2.5) | 0.202 | -3.7(-9.4 to 2.5) | 0.202 |
|  |  |  |  |  |  |  | 2016-2019 | 32.0(-15.1 to 105.3) | 0.167 |  |  |  |  |  |  |  |  |
| Small cell carcinoma, NOS | 2010-2019 | -11.6(-20.9 to -1.2) | 0.034 | -11.6(-20.9 to -1.2) | 0.034 |  | 2010-2019 | -13.4(-23.5 to -2.0) | 0.028 | -13.4(-23.5 to -2.0) | 0.028 |  | 2010-2019 | -6.9(-12.6 to -0.9) | 0.030 | -6.9(-12.6 to -0.9) | 0.030 |
| Squamous cell carcinoma, NOS | 2010-2019 | 0.3(-0.0 to 0.6) | 0.081 | 0.3(-0.0 to 0.6) | 0.081 |  | 2010-2019 | 0.2(-0.1 to 0.4) | 0.195 | 0.2(-0.1 to 0.4) | 0.195 |  | 2010-2019 | 0.4(0.0 to 0.9) | 0.051 | 0.4(0.0 to 0.9) | 0.051 |
| Lymphoepithelial carcinoma |  |  |  |  |  |  | 2010-2019 | 2.6(-11.3 to 18.7) | 0.693 | 2.6(-11.3 to 18.7) | 0.693 |  | 2010-2019 | -13.1(-20.5 to -5.0) | 0.007 | -13.1(-20.5 to -5.0) | 0.007 |
| Adenocarcinoma, NOS |  |  |  |  |  |  | 2010-2013 | 46.6(-25.7 to 189.2) | 0.207 | 6.8(-11.2 to 28.4) | 0.487 |  | 2010-2019 | 1.5(-5.4 to 8.9) | 0.634 | 1.5(-5.4 to 8.9) | 0.634 |
|  |  |  |  |  |  |  | 2013-2019 | -8.9(-19.9 to 3.6) | 0.121 |  |  |  |  |  |  |  |  |
| **Gastric cance**r |  |  |  |  |  |  |  |  |  |  |  |  |  |  |  |  |  |
| Other | 2010-2019 | 5.3(-2.7 to 13.9) | 0.170 | 5.3(-2.7 to 13.9) | 0.170 |  | 2010-2019 | -4.5(-9.8 to 1.1) | 0.101 | -4.5(-9.8 to 1.1) | 0.101 |  | 2010-2019 | -4.8(-12.1 to 3.1) | 0.192 | -4.8(-12.1 to 3.1) | 0.192 |
| Carcinoma, NOS | 2010-2019 | -9.6(-16.5 to -2.2) | 0.019 | -9.6(-16.5 to -2.2) | 0.019 |  | 2010-2019 | -7.2(-11.7 to -2.4) | 0.009 | -7.2(-11.7 to -2.4) | 0.009 |  | 2010-2015 | -22.2(-32.8 to -10.0) | 0.007 | -5.1(-14.5 to 5.3) | 0.323 |
|  |  |  |  |  |  |  |  |  |  |  |  |  | 2015-2019 | 21.7(-5.0 to 55.9) | 0.097 |  |  |
| Adenocarcinoma, NOS | 2010-2017 | -0.8(-1.2 to -0.3) | 0.009 | -0.1(-0.8 to 0.5) | 0.690 |  | 2010-2019 | 0.1(-0.2 to 0.3) | 0.517 | 0.1(-0.2 to 0.3) | 0.517 |  | 2010-2016 | 0.8(0.1 to 1.6) | 0.039 | 0.2(-0.4 to 0.8) | 0.543 |
|  | 2017-2019 | 2.1(-1.3 to 5.7) | 0.174 |  |  |  |  |  |  |  |  |  | 2016-2019 | -1.1(-3.0 to 0.9) | 0.224 |  |  |
| Carcinoid tumor, malignant | 2010-2017 | 31.5(13.6 to 52.3) | 0.005 | 11.7(-3.7 to 29.5) | 0.145 |  | 2010-2015 | 27.0(5.5 to 53.0) | 0.021 | 4.2(-6.8 to 16.4) | 0.471 |  | 2010-2019 | -0.6(-10.9 to 10.9) | 0.903 | -0.6(-10.9 to 10.9) | 0.903 |
|  | 2017-2019 | -37.0(-69.0 to 28.0) | 0.155 |  |  |  | 2015-2019 | -18.7(-35.5 to 2.4) | 0.070 |  |  |  |  |  |  |  |  |
| Mucinous adenocarcinoma | 2010-2019 | -0.6(-5.7 to 4.8) | 0.805 | -0.6(-5.7 to 4.8) | 0.805 |  | 2010-2019 | -4.3(-10.4 to 2.2) | 0.158 | -4.3(-10.4 to 2.2) | 0.158 |  | 2010-2012 | -30.6(-74.5 to 88.6) | 0.390 | -4.6(-20.3 to 14.3) | 0.610 |
|  |  |  |  |  |  |  |  |  |  |  |  |  | 2012-2019 | 4.5(-5.8 to 16.0) | 0.326 |  |  |
| Signet ring cell carcinoma | 2010-2019 | 0.8(-5.0 to 6.9) | 0.770 | 0.8(-5.0 to 6.9) | 0.770 |  |  | 2.8(-4.8 to 11.1) | 0.425 | 2.8(-4.8 to 11.1) | 0.425 |  | 2010-2019 | -0.6(-6.7 to 5.9) | 0.836 | -0.6(-6.7 to 5.9) | 0.836 |
| Stromal sarcoma | 2010-2019 | 4.6(-3.7 to 13.7) | 0.247 | 4.6(-3.7 to 13.7) | 0.247 |  | -- | -- | -- |  |  |  | 2010-2019 | 8.6(2.2 to 15.4) | 0.014 | 8.6(2.2 to 15.4) | 0.014 |

**Supplementary Table 10** Changing trajectories of histologic subtypes for esophageal cancer, by stage group, 2010–2019.

|  | Period | APC (95%CI) | *P* value | AAPC (95%CI) | *P* value |
| --- | --- | --- | --- | --- | --- |
| **Stage I** |  |  |  |  |  |
| Carcinoma, NOS |  |  |  |  |  |
| Small cell carcinoma, NOS |  |  |  |  |  |
| Squamous cell carcinoma, NOS | 2010-2019 | 0.7(0.1 to 1.3) | 0.041 | 0.7(0.1 to 1.3) | 0.041 |
| Lymphoepithelial carcinoma | 2010-2019 | -4.0(-12.3 to 5.1) | 0.326 | -4.0(-12.3 to 5.1) | 0.326 |
| Adenocarcinoma, NOS |  |  |  |  |  |
| Other |  |  |  |  |  |
| **Stage II** |  |  |  |  |  |
| Carcinoma, NOS |  |  |  |  |  |
| Small cell carcinoma, NOS | 2010-2019 | -5.5(-13.9 to 3.9) | 0.206 | -5.5(-13.9 to 3.9) | 0.206 |
| Squamous cell carcinoma, NOS | 2010-2019 | -0.0(-0.4 to 0.4) | 0.953 | -0.0(-0.4 to 0.4) | 0.953 |
| Lymphoepithelial carcinoma |  |  |  |  |  |
| Adenocarcinoma, NOS |  |  |  |  |  |
| Other | 2010-2019 | 9.0(-4.1 to 23.7) | 0.159 | 9.0(-4.1 to 23.7) | 0.159 |
| **Stage III** |  |  |  |  |  |
| Carcinoma, NOS | 2010-2012 | -26.4(-93.8 to 769.1) | 0.762 | -3.6(-36.7 to 46.8) | 0.863 |
|  | 2012-2019 | 4.1(-3.6 to 12.3) | 0.235 |  |  |
| Small cell carcinoma, NOS | 2010-2019 | -4.0(-11.7 to 4.4) | 0.297 | -4.0(-11.7 to 4.4) | 0.297 |
| Squamous cell carcinoma, NOS | 2010-2019 | 0.1(-0.2 to 0.4) | 0.532 | 0.1(-0.2 to 0.4) | 0.532 |
| Lymphoepithelial carcinoma | 2010-2019 | -14.3(-21.7 to -6.3) | 0.004 | -14.3(-21.7 to -6.3) | 0.004 |
| Adenocarcinoma, NOS |  |  |  |  |  |
| Other | 2010-2019 | 1.5(-8.2 to 12.3) | 0.739 | 1.5(-8.2 to 12.3) | 0.739 |
| **Stage IV** |  |  |  |  |  |
| Carcinoma, NOS |  |  |  |  |  |
| Small cell carcinoma, NOS | 2010-2019 | -4.5(-16.9 to 9.8) | 0.469 | -4.5(-16.9 to 9.8) | 0.469 |
| Squamous cell carcinoma, NOS | 2010-2019 | 1.0(0.1 to 2.0) | 0.046 | 1.0(0.1 to 2.0) | 0.046 |
| Lymphoepithelial carcinoma |  |  |  |  |  |
| Adenocarcinoma, NOS |  |  |  |  |  |
| Other |  |  |  |  |  |

**Supplementary Table 11** Changing trajectories of subsites for esophageal cancer and gastric cancer, by sex group, 2010–2019

|  | Male | | | | |  | Female | | | | |
| --- | --- | --- | --- | --- | --- | --- | --- | --- | --- | --- | --- |
|  | Period | APC (95%CI) | *P* value | AAPC (95%CI) | P value |  | Period | APC (95%CI) | *P* value | AAPC (95%CI) | *P* value |
| **Esophageal cancer** |  |  |  |  |  |  |  |  |  |  |  |
| Upper third of oesophagus | 2010-2013 | -15.1(-31.2 to 4.9) | 0.103 | -4.7(-11.2 to 2.4) | 0.190 |  | 2010-2019 | -3.4(-7.9 to 1.3) | 0.132 | -3.4(-7.9 to 1.3) | 0.132 |
|  | 2013-2019 | 1.0(-7.9 to 10.8) | 0.786 |  |  |  |  |  |  |  |  |
| Middle third of oesophagus | 2010-2019 | -6.9(-9.3 to -4.4) | <0.001 | -6.9(-9.3 to -4.4) | <0.001 |  | 2010-2013 | -12.3(-20.0 to -3.8) | 0.015 | -4.4(-7.3 to -1.3) | 0.005 |
|  |  |  |  |  |  |  | 2013-2019 | -0.1(-4.2 to 4.1) | 0.936 |  |  |
| Lower third of oesophagus | 2010-2019 | 2.5(0.1 to 4.9) | 0.041 | 2.5(0.1 to 4.9) | 0.041 |  | 2010-2019 | 2.4(-4.3 to 9.5) | 0.438 | 2.4(-4.3 to 9.5) | 0.438 |
| **Gastric cancer** |  |  |  |  |  |  |  |  |  |  |  |
| Cardia | 2010-2014 | 5.9(-1.4 to 13.8) | 0.095 | -0.9(-4.0 to 2.4) | 0.605 |  | 2010-2012 | 15.2(-16.9 to 59.8) | 0.315 | -4.1(-9.9 to 2.0) | 0.186 |
|  | 2014-2019 | -6.0(-10.0 to -1.1) | 0.026 |  |  |  | 2012-2019 | -9.0(-13.2 to -4.6) | 0.004 |  |  |
| Non- cardia | 2010-2014 | -3.1(-6.3 to 0.2) | 0.059 | 0.4(-1.0 to 1.9) | 0.553 |  | 2010-2012 | -2.9(-10.4 to 5.4) | 0.401 | 0.8(-0.7 to 2.3) | 0.280 |
|  | 2014-2019 | 3.3(1.3 to 5.5) | 0.009 |  |  |  | 2012-2019 | 1.9(1.0 to 2.8) | 0.003 |  |  |
| Fundus of stomach | 2010-2019 | -0.3(-7.0 to 7.0) | 0.934 | -0.3(-7.0 to 7.0) | 0.934 |  | 2010-2015 | 9.2(-28.3 to 66.3) | 0.615 | 4.1(-14.9 to 27.4) | 0.695 |
|  |  |  |  |  |  |  | 2015-2019 | -1.9(-25.7 to 29.6) | 0.868 |  |  |
| Body of stomach | 2010-2019 | -0.6(-3.7 to 2.6) | 0.682 | -0.6(-3.7 to 2.6) | 0.682 |  | 2010-2017 | 4.8(1.2 to 8.6) | 0.019 | 0.3(-4.2 to 5.1) | 0.891 |
|  |  |  |  |  |  |  | 2017-2019 | -13.9(-32.4 to 9.8) | 0.174 |  |  |
| Gastric antrum | 2010-2017 | 2.5(0.6 to 4.4) | 0.018 | -0.8(-3.2 to 1.7) | 0.537 |  | 2010-2013 | -4.4(-10.0 to 1.6) | 0.086 | -2.0(-3.9 to -0.1) | 0.036 |
|  | 2017-2019 | -11.4(-22.5 to 1.2) | 0.067 |  |  |  | 2013-2017 | 5.9(-0.1 to 12.3) | 0.051 |  |  |
|  |  |  |  |  |  |  | 2017-2019 | -13.1(-22.8 to -2.3) | 0.036 |  |  |

**Supplementary Table 12** Changing trajectories of histologic subtypes for gastric cancer, by stage group, 2010–2019.

|  | Period | APC (95%CI) | *P* value | AAPC (95%CI) | *P* value |
| --- | --- | --- | --- | --- | --- |
| **Stage I** |  |  |  |  |  |
| Carcinoma, NOS |  | -- | -- | -- | -- |
| Adenocarcinoma, NOS | 2010-2013 | 3.0(0.6 to 5.4) | 0.023 | 0.9(0.2 to 1.6) | 0.011 |
|  | 2013-2019 | -0.1(-0.8 to 0.5) | 0.600 |  |  |
| Carcinoid tumor, malignant |  | -- | -- | -- | -- |
| Mucinous adenocarcinoma |  | -- | -- | -- | -- |
| Signet ring cell carcinoma | 2010-2019 | -5.5(-11.1 to 0.5) | 0.066 | -5.5(-11.1 to 0.5) | 0.066 |
| Stromal sarcoma |  | -- | -- | -- | -- |
| Other | 2010-2019 | -4.1(-14.8 to 7.9) | 0.434 | -4.1(-14.8 to 7.9) | 0.434 |
| **Stage II** |  |  |  |  |  |
| Carcinoma, NOS |  | -- | -- | -- | -- |
| Adenocarcinoma, NOS | 2010-2019 | -0.2(-0.8 to 0.4) | 0.431 | -0.2(-0.8 to 0.4) | 0.431 |
| Carcinoid tumor, malignant | 2010-2017 | 19.3(10.1 to 29.1) | 0.002 | -4.1(-23.4 to 20.0) | 0.715 |
|  | 2017-2019 | -55.3(-87.7 to 63.2) | 0.171 |  |  |
| Mucinous adenocarcinoma | -- | -- | -- | -- |  |
| Signet ring cell carcinoma | 2010-2019 | 5.0(-6.0 to 17.4) | 0.339 | 5.0(-6.0 to 17.4) | 0.339 |
| Stromal sarcoma |  | -- | -- | -- | -- |
| Other | 2010-2019 | -7.3(-15.4 to 1.6) | 0.094 | -7.3(-15.4 to 1.6) | 0.094 |
| **Stage III** |  |  |  |  |  |
| Carcinoma, NOS | 2010-2017 | 3.8(-7.9 to 17.0) | 0.457 | 17.0(5.9 to 29.2) | 0.002 |
|  | 2017-2019 | 77.5(17.7 to 167.6) | 0.016 |  |  |
| Adenocarcinoma, NOS | 2010-2019 | -0.4(-0.7 to -0.1) | 0.034 | -0.4(-0.7 to -0.1) | 0.034 |
| Carcinoid tumor, malignant | -- | -- | -- | -- | -- |
| Mucinous adenocarcinoma | 2010-2019 | -5.1(-9.9 to -0.1) | 0.046 | -5.1(-9.9 to -0.1) | 0.046 |
| Signet ring cell carcinoma | 2010-2017 | 20.8(4.5 to 39.7) | 0.020 | 2.0(-18.0 to 26.9) | 0.859 |
|  | 2017-2019 | -43.6(-82.7 to 84,4) | 0.269 |  |  |
| Stromal sarcoma |  | -- | -- | -- | -- |
| Other |  | -- | -- | -- | -- |
| **Stage IV** |  |  |  |  |  |
| Carcinoma, NOS | 2010-2019 | 4.0(-0.6 to 8.8) | 0.084 | 4.0(-0.6 to 8.8) | 0.084 |
| Adenocarcinoma, NOS | 2010-2019 | -0.7(-1.1 to -0.3) | 0.004 | -0.7(-1.1 to -0.3) | 0.004 |
| Carcinoid tumor, malignant | -- | -- | -- | -- | -- |
| Mucinous adenocarcinoma | 2010-2019 | 2.1(-7.0 to 12.0) | 0.625 | 2.1(-7.0 to 12.0) | 0.625 |
| Signet ring cell carcinoma | 2010-2019 | 3.8(-2.1 to 9.9) | 0.179 | 3.8(-2.1 to 9.9) | 0.179 |
| Stromal sarcoma |  | -- | -- | -- | -- |
| Other | 2010-2019 | 6.5(-3.1 to 17.2) | 0.163 | 6.5(-3.1 to 17.2) | 0.163 |
